# *TET2* deficiency promotes MDS-associated leukemogenesis

**DOI:** 10.1038/s41408-022-00739-w

**Published:** 2022-10-04

**Authors:** Feiteng Huang, Jie Sun, Wei Chen, Lei Zhang, Xin He, Haojie Dong, Yuhui Wu, Hanying Wang, Zheng Li, Brian Ball, Samer Khaled, Guido Marcucci, Ling Li

**Affiliations:** 1 Department of Hematological Malignancies Translational Science, Gehr Family Center for Leukemia Research, Hematologic Malignancies and Stem Cell Transplantation Institute, Beckman Research Institute, City of Hope Medical Center, Duarte, CA 91010 USA; 2 Department of Hematology, Sir Run Run Shaw Hospital, School of Medicine, Zhejiang University, 310016 Hangzhou, China; 3 The Integrative Genomics Core, Beckman Research Institute, City of Hope Medical Center, Duarte, CA 91010 USA; 4 Department of Hematology and Hematopoietic Cell Transplantation (HCT), Beckman Research Institute, City of Hope Medical Center, Duarte, CA 91010 USA

**Keywords:** Myelodysplastic syndrome, Cancer stem cells

Dear Editor,

Myelodysplastic syndrome (MDS) is a group of clonal hematopoietic disorders that frequently progress to acute myeloid leukemia (AML) [1]. However, mechanisms underlying such transformation are not yet fully understood. *TET2* is one of the most frequently mutated genes in myeloid malignancies [2]. We previously demonstrated that post-translational modification of TET2 protein led to DNA hypermethylation and dysregulated gene expression in MDS hematopoietic stem and progenitor cells (HSPCs), conferring a clonal advantage [3]. *TET2* down-regulation was also seen during MDS progression [4]. Herein, we retrospectively analyzed GEO datasets including AML or MDS sample cohort. We found that lower *TET2* levels seen in high-risk MDS (HR-MDS) were closely associated with shorter survival (Fig. S1A–C) [5]. Moreover, relative to those with wild type (WT) *TET2*, AML patients harboring *TET2* mutations exhibited a lower survival rate and a higher likelihood of AML secondary to MDS or MPN (Fig. S1D, E) [6]. Collectively, these observations prompted us to assess TET2 function in leukemia transformation of MDS.

To do so, we used a *Nup98-HoxD13* (*NHD13*) transgenic mouse model, in which ~30% of mice develop AML. Interestingly, TET2 levels were lower in c-kit^+^ bone marrow (BM) cells of leukemia-transformed *NHD13* mice relative to age-matched *NHD13* mice, which developed MDS exclusively (Fig. S1F, G), suggesting transformation linked to TET2 downregulation. Thus, we crossed *Tet2* conditional knockout (KO, *Tet2*^fl/fl^/*Mx1*-Cre) or corresponding control (*Tet2*^fl/fl^) mice with *NHD13* mice and monitored leukemia development following poly(I:C) treatment on both genotypes (Fig. S1H, I). Notably, *Tet2* deletion shortened median survival of *NHD13* mice (Fig. 1A). Within 30 weeks, 5 of the 10 mice from the *NHD13*/*Tet2*-KO cohort developed AML, while none of the *NHD13*/*Tet2*-WT mice exhibited signs of leukemia (Table S1). Specifically, leukemic *NHD13*/*Tet2*-KO mice showed increased white blood cell (WBC) counts, splenomegaly and hyper-cellularity in BM, while age-matched *NHD13*/*Tet2*-WT mice exhibited only cytopenia (Figs. 1B and S1J–L). *NHD13*/*Tet2*-KO mice showed increases in the c-kit^+^ subset and blasts in BM compared to *NHD13*/*Tet2*-WT mice (Figs. 1C, D and S1M). Moreover, secondary recipients also developed AML following transplant of leukemic *NHD13*/*Tet2*-KO BM cells (Fig. S1N, O).

**Fig. 1:**
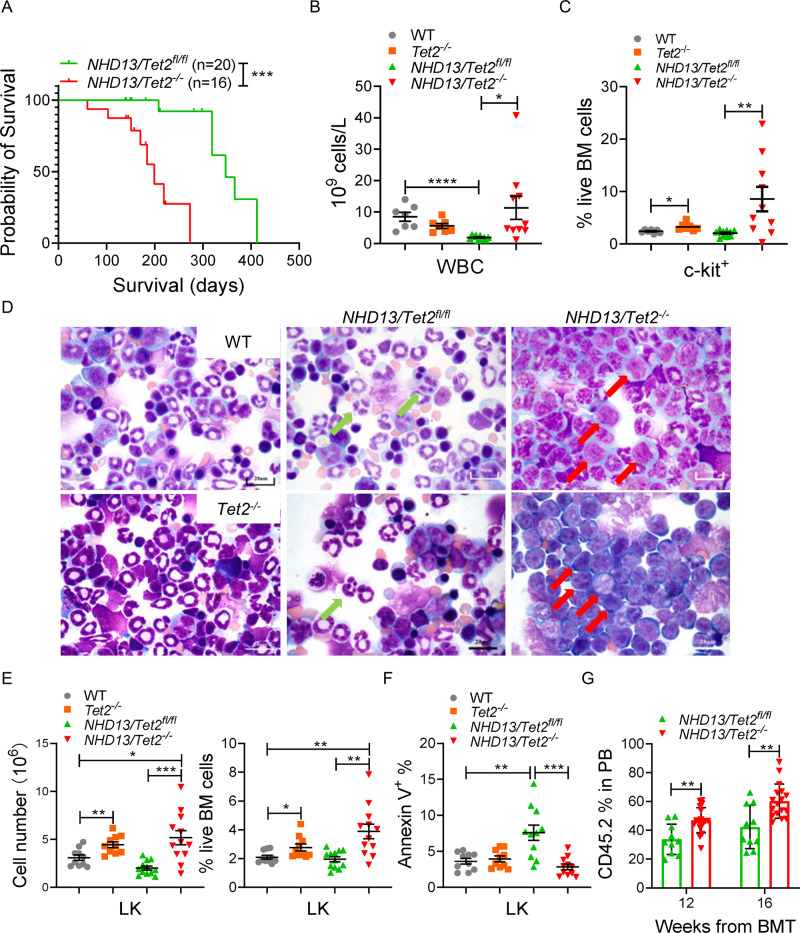
*Tet2* deficiency expands the stem/progenitor pool and accelerates leukemia transformation in a murine model of MDS. **A** Survival of *NHD13*/*Tet2*-WT (*n* = 20; median survival, 347 days) and *NHD13*/*Tet2*-KO (*n* = 16; median survival, 199 days) mice. **B** WBC count of WT, *Tet2*-KO, *NHD13*, and *NHD13*/*Tet2*-KO mice (30-weeks-old). **C** Frequencies of BM c-kit^+^ cells from indicated mice at 30-weeks-old. **D** Wright–Giemsa staining of BM cells from indicated mice. Green arrows: dysplastic cells; red arrows: blast cells; scale bars, 20 μm. **E** Total cell number and percentage of LK subsets in BM of indicated primary mice at a pre-leukemic stage (20-weeks-old). **F** Apoptosis of LK population in the BM of indicated mice based on Annexin V staining. **G** Lethally-irradiated mice were transplanted with 2 × 10^5^ LK cells from pre-leukemic *NHD13*/*Tet2*-WT (*n* = 10) or *NHD13*/*Tet2*-KO (*n* = 18) mice plus 2 × 10^5^ unfractionated WT support cells. Shown is chimerism of donor-derived cells (CD45.2^+^) in PB of recipient mice at different time points. *P < 0.05; **P < 0.01; ***P < 0.001, ****P < 0.0001.

*NHD13* transgenic mice were characterized by *HoxA9* elevation [7]. Thus we evaluated TET2 function in MDS or AML patients that showed differences in *HOXA9* expression. While *HOXA9* or *TET2* levels alone did not predict prognosis of the entire MDS population, the *HOXA9*^high^/*TET2*^low^ combination predicted shorter survival relative to those with *HOXA9*^low^/*TET2*^high^ (Fig. S1P–R). Moreover, *TET2* mutation also predicted shorter overall survival in the *HOXA9*^high^ AML population (Fig. S1S, T). We then transduced *Tet2*-WT or *Tet2*-KO BM cells with *HoxA*9 and transplanted the cells into recipient mice to monitor leukemia development. We observed that 6 of 8 *HoxA9*/*Tet2*-WT recipients survived up to 200 days, while all 8 recipients of *HoxA9*/*Tet2*-KO cells developed lethal AML, starting at day 62 (Fig. S1U, V), suggesting that *Tet2* deletion promotes AML transformation and in agreement with outcomes seen in *NHD13/Tet2-*KO mice.

To define mechanisms underlying MDS progression, we evaluated the *Tet2*-KO vs. *Tet2*-WT *NHD13* BM compartment at a pre-leukemic stage (20-weeks-old). At that time point, neither genotype showed signs of leukemia (Fig. S2A), but *Tet2* deletion increased the number of monocytes in BM of *NHD13* mice (Fig. S2B–D). Importantly, BM cells from *NHD13/Tet2-KO* mice showed an increase in the Lin^-^c-kit^+^Sca-1^−^ (LK) population relative to those of *Tet2*-WT *NHD13* mice, whereas the Lin^-^c-kit^+^Sca-1^+^ (LSK) population was unchanged by *Tet2* deletion (Figs. 1E and S2E). Increases in the LK subset are likely due to decreased apoptosis following *Tet2*-KO (Fig. 1F). Within the LK subset, we observed increases in common myeloid progenitors (CMPs) and granulocyte-monocyte progenitors (GMPs) in *NHD13/Tet2-KO* mice (Fig. S2F, G). Moreover, *Tet2* loss did not alter the cell cycle of c-kit^+^ cells (Fig. S2H) or that of the LK subset (not shown). Colony-forming cell (CFC) assays revealed that *NHD13*/*Tet2*-KO BM cells formed colonies in the absence of cytokines (Fig. S2I). In the presence of cytokines, *Tet2*-KO cells exhibited a slightly higher CFC number than did *Tet2*-WT cells, the difference was further amplified in the context of *NHD13* (Fig. S2J, K). *Tet2*-KO cells also exhibited higher replating capacity than did *Tet2*-WT cells (Fig. S2L). We next transplanted LK cells (CD45.2^+^) from pre-leukemic *NHD13*/*Tet2*-KO or corresponding control *NHD13* mice into lethally-irradiated secondary recipients to assess leukemogenicity. As expected, *NHD13*/*Tet2*-KO cell transplantation increased the percentage of CD45.2^+^ cells and WBCs in peripheral blood (PB) relative to *NHD13*/*Tet2*-WT cells (Figs. 1G and S2M, N). By 16 weeks post-transplantation, 14 of 18 *NHD13*/*Tet2*-KO recipients developed AML and exhibited increased numbers of c-kit^+^ cells and blasts in PB (Fig. S2O, P). Notably, *NHD13*/*Tet2*-KO transplants showed shorter survival than *NHD13*/*Tet2*-WT transplants (Fig. S2Q). Moreover, we also analyzed the transplants using donor c-kit^+^ cells (CD45.2^+^) from WT or *Tet2*-KO mice (Fig. S2R, S) and observed that recipients from both genotypes survived up to 24 weeks following transplantation, with no signs of leukemic transformation (data not shown). Collectively, these results indicate that *Tet2*-KO-mediated leukemogenesis is associated with expansion of the MDS HSPC (LK subset) pool.

*Tet2* loss in HSPCs can lead to hypermutagenicity [8]. To evaluate these outcomes, we performed whole-exome sequencing of c-kit^+^ cells from pre-leukemic *NHD13*/*Tet2*-KO vs. matched *NHD13*/*Tet2*-WT mice. Relative to *NHD13*/*Tet2*-WT mice, we observed 271 newly acquired alterations and 199 alterations with increased variant allele frequency (VAF, fold-change >1.5) in *NHD13*/*Tet2*-KO mice (Fig. S3A and Table S2). KEGG analysis of these alterations (271 + 199) in *NHD13*/*Tet2*-KO mice revealed significant enrichment of genes related to cancer and signaling pathways (Fig. S3B). We next focused on the top 70 altered genes (VAF fold change>2, *P* < 0.05) and ranked them based on association with AML prognosis (http://precog.stanford.edu) (Table S3). Accordingly, we selected *Setd2*, *Arih2* and *Tet3* for further study due to their higher average VAF (VAF > 50%) and mutation ratio (mutation ratio >0.5) (Fig. 2A). Pairwise cooperativeness distribution of these genes showed that *Tet3* mutation had the highest pairing associations with other mutations (Fig. S3C). To assess the impact of *Setd2*, *Arih2*, and *Tet3* loss-of-function mutations, we transduced *NHD13* c-kit^+^ cells with shRNA targeting each gene individually and performed a CFC assay (Fig. S3D). In the first plating, among all genes analyzed, *Arih2* knockdown (KD) promoted the greatest increase in colony and cell number of *NHD13* c-kit^+^ cells (Fig. 2B). However, *Arih2*-KD cells did not show higher replating capacity (Fig. S3E), indicating that *Arih2* loss merely impacts proliferation.

**Fig. 2:**
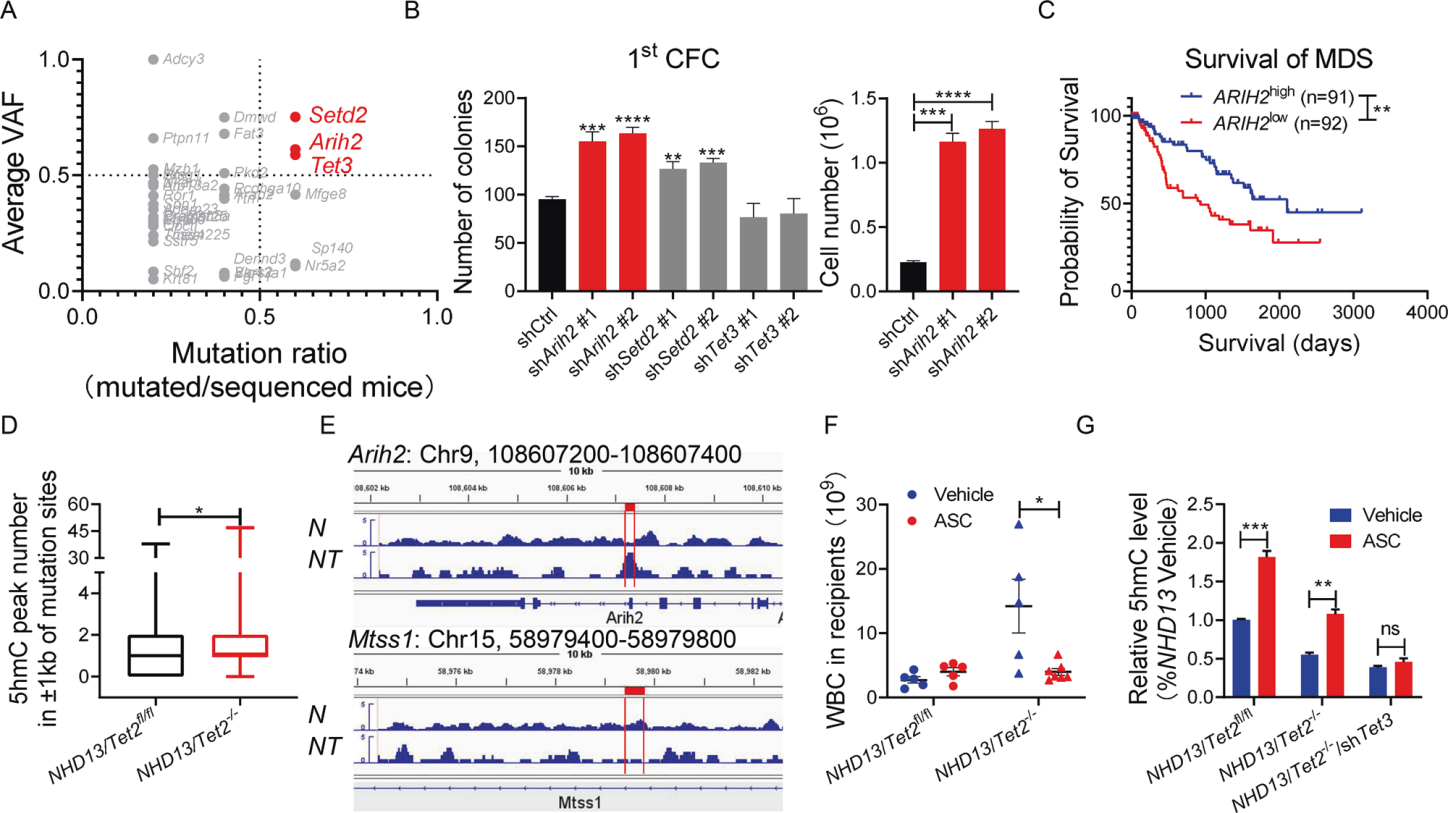
Vitamin C treatment mimics *Tet2* restoration and blocks leukemogenesis. **A** Depicted are 37 genes identified from PRECOG based on two conditions (VAF > 50%, mutation ratio >0.5). Red dots represent preferential enrichment of mutations associated with AML poor prognosis. **B** c-kit^+^ BM cells from *NHD13* mice were transduced with shRNA targeting indicated genes and then plated for CFC. Shown are colony number (left) and cell number (right) of sh*Arih2* or shCtrl cells after first plating. **C** Overall survival of total MDS patients, stratified by *ARIH2* expression levels. **D** 5hmC peaks around mutation sites (±1 kb) were counted in *Tet2*-WT or *Tet2*-KO *NHD13* mice (*P* = 0.0265, paired *t* test). **E** Representative University of California Santa Cruz (UCSC) tracks showing 5hmC peaks (red boxes) associated with *Arih2* (Chr9: 108607200–108607400) and *Mtss1* (Chr15: 58979400–58979800) loci. Tracks showing 5hmC enrichment were from biological replicates of BM c-kit^+^ cells from *NHD13*/*Tet2*-WT (N) or *NHD13*/*Tet2*-KO (NT) mice. **F** Vitamin C treatment of mice reconstituted with *NHD13*/*Tet2*^fl/fl^ and *NHD13*/*Tet2*^fl/fl^/*Mx1*-Cre BM. *Tet2* was deleted by injection of poly(I:C) at 6 weeks post-transplant. Mice were intraperitoneally injected with normal saline (vehicle) or ascorbate (ASC, 4 g/kg), 5 days a week for 16 weeks. WBC counts were monitored at 24 weeks post-transplant. **G** *NHD13*/*Tet2*^fl/fl^, *NHD13*/*Tet2*^-/-^, and *NHD13*/*Tet2*^-/-^/sh*Tet3* BM c-kit^+^ cells were treated with vehicle or ASC (0.25 mM) for 3 days and relative 5hmC levels in total DNA were measured by ELISA. *P < 0.05; **P < 0.01; ***P < 0.001; ****P < 0.0001.

To define *ARIH2* function in leukemogenesis, we retrospectively analyzed GEO datasets and observed lower *ARIH2* expression associated with shorter survival in MDS and AML patients (Figs. 2C and S4A). Moreover, *SETD2* or *TET3* levels alone were not associated with MDS prognosis (Fig. S4B, C). Genetic deletion of endogenous *ARIH2* significantly increased FGFR1 protein levels as well as downstream ERK, STAT5 and AKT activity in FGFR1-proficient K562 cells (Fig. S4D). Notably, in *ARIH2*-KD K562 cells, overexpression of *ARIH2*^WT^ down-regulated FGFR1 protein and its downstream effectors, while overexpression of *ARIH2*^K441N^ (analogous to the mouse *Arih2*^K440N^) did not, and cells harboring the mutant exhibited a growth advantage relative to *ARIH2*^WT^ cells (Fig. S4E, F). Similarly, *ARIH2* overexpression also inhibited growth of MDS-L/*TET2*-KD cells relative to MOCK controls (Fig. S4G–I). To extend this analysis in vivo, we injected *ARIH2*^WT^ or MOCK MDS-L/*TET2*-KD cells into NSGS mice and observed that *ARIH2*^WT^ overexpression significantly decreased MDS-L/*TET2*-KD cell engraftment in BM relative to MOCK controls (Fig. S4J).

To assess whether *TET2* deficiency underlies increased mutation frequency, we analyzed GEO datasets and found that MDS or AML patients with *TET2* mutations harbor a greater number of mutational events (excluding *TET2* itself) compared to those with *TET2*-WT (Fig. S5A) [9–11]. Additionally, *ASXL1* mutation also predicted a higher mutation frequency in MDS and AML patients, whereas other mutations analyzed did not (Fig. S5B–D). Further analysis of tumor mutational burden (TMB) profiles from The Cancer Genome Atlas (TCGA) leukemia cohort showed that patients with mutations in either *TET2* or *ASXL1* displayed significantly higher TMB levels than those with respective WT genes, whereas mutations in *DNMT3A*, *NRAS* or *FLT3* alone did not predict higher TMB (Fig. S5E, F). An HPRT assay showed that *TET2*-deficient K562 cells exhibited an increase in HPRT spontaneous mutation frequency relative to controls (Fig. S5G).

The hMeDIP-seq analysis of murine MDS HSPCs indicated that *Tet2* deletion decreased overall 5hmC levels (Fig. S5H). Moreover, correlation of 5hmC enrichment with mutation loci showed a greater number of 5hmC peaks at mutation sites in *NHD13*/*Tet2*-KO c-kit^+^ compared to *NHD13* c-kit^+^ cells (Fig. 2D). Specifically, *NHD13*/*Tet2*-KO cells exhibited a 5hmC peak enriched at the *Arih2* locus but decreased 5hmC enrichment at the enhancer of *Mtss1*, a reported *Tet2* target locus (Fig. 2E). Moreover, hMeDIP-qPCR analyses also revealed that *Tet2* deletion increased 5hmC enrichment at mutation sites of *Arih2*, *Setd2* and *Tet3* (Fig. S5I), in agreement with reports of others that *TET2* loss may enrich 5hmC peaks at mutation loci [8].

Given that vitamin C treatment mimics effects of *Tet2*/*Tet3* restoration [12], we treated transplant mice reconstituted *NHD13* or *NHD13*/*Tet2-*KO BM with either normal saline (vehicle) or ascorbate (ASC, the dominant form of vitamin C). Relative to vehicle-treated *NHD13/Tet2-KO* recipients, ASC treatment in *NHD13/Tet2-KO* mice significantly decreased WBC counts and the frequency of c-kit^+^ BM cells (Figs. 2F and S6A). At 24 weeks post-transplant, 5 of the 7 *NHD13*/*Tet2*-KO recipients from the vehicle cohort developed AML, while none of the ASC-treated mice showed signs of leukemia (Fig. S6B). Notably, vitamin C effects in BM cells from *NHD13*/*Tet2*-KO mice were partially dependent on *Tet3*, as 5hmC increases seen in cells from *NHD13*/*Tet2*-KO mice were markedly attenuated upon *Tet3* KD (Fig. 2G). Moreover, an HPRT assay in K562/*TET2*-KD cells confirmed that vitamin C treatment prevented mutagenicity induced by *TET2* deficiency (Fig. S6C). Finally, CFC assays with purified blasts (CD34^+^) from two *TET2* mutant high-risk MDS patients (Table S4) showed that vitamin C treatment decreased CFC of MDS HSPCs, while sparing healthy CD34^+^ cells (Fig. S6D).

In summary, our results indicate that TET2 activity prevents further transformation of MDS HSPCs by decreasing the occurrence of secondary mutations, and that pharmacological enhancement of TET activity may represent an optimal strategy to block MDS malignant transformation.

### Supplementary information

Supplementary data

## Data Availability

The accession number for the whole-exome sequencing of c-kit^+^ cells from *NHD13*/*Tet2*-KO or *NHD13*/*Tet2*-WT mice reported in this paper is GEO: GSE213530. The accession number for the hMeDIP-seq data is GEO: GSE213591. All other remaining data are available on request.
