# Correction: *TET2* deficiency promotes MDS-associated leukemogenesis

**DOI:** 10.1038/s41408-023-00952-1

**Published:** 2023-11-28

**Authors:** Feiteng Huang, Jie Sun, Wei Chen, Lei Zhang, Xin He, Haojie Dong, Yuhui Wu, Hanying Wang, Zheng Li, Brian Ball, Samer Khaled, Guido Marcucci, Ling Li

**Affiliations:** 1 Department of Hematological Malignancies Translational Science, Gehr Family Center for Leukemia Research, Hematologic Malignancies and Stem Cell Transplantation Institute, Beckman Research Institute, City of Hope Medical Center, Duarte, CA 91010 USA; 2 Department of Hematology, Sir Run Run Shaw Hospital, School of Medicine, Zhejiang University, 310016 Hangzhou, China; 3 The Integrative Genomics Core, Beckman Research Institute, City of Hope Medical Center, Duarte, CA 91010 USA; 4 Department of Hematology and Hematopoietic Cell Transplantation (HCT), Beckman Research Institute, City of Hope Medical Center, Duarte, CA 91010 USA

**Keywords:** Myelodysplastic syndrome, Cancer stem cells

Correction to: *Blood Cancer Journal* 10.1038/s41408-022-00739-w, published online 04 October 2022

In this article, Acknowledgements section was incorrectly given. It should have read as below.

This work was supported in part by National Institutes of Health, National Heart, Lung, and Blood Institute grant R01 HL141336, R01 CA248149, a Research Scholar grant (RSG-19-036-01-LIB) from the American Cancer Society, the Gehr Family Center for Leukemia Research (LL) and the Vera and Joseph Dresner Foundation for established investigator award (LL and GM).

The original article has been corrected.

