# Supplementary material for: TET2 deficiency promotes MDS-associated leukemogenesis
Source: Blood Cancer J. 2022 Oct 4;12(10):141. doi: 10.1038/s41408-022-00739-w (PMC9532431; doi:10.1038/s41408-022-00739-w)
Supplement: Supplementary file 1 — Supplementary data [file 41408_2022_739_MOESM1_ESM.pdf]

Supplementary Information

TET2 deficiency promotes MDS-associated leukemogenesis

Feiteng Huang[1,2], Jie Sun[1], Wei Chen[3], Lei Zhang[1], Xin He[1], Haojie Dong[1], Yuhui Wu[1], Hanying Wang[1], Zheng Li[1], Brian Ball[4], Samer Khaled[4], Guido Marcucci[1,4], Ling Li[1]

[1]Department of Hematological Malignancies Translational Science, Gehr Family Center for Leukemia Research, Hematologic Malignancies and Stem Cell Transplantation Institute, Beckman Research Institute, City of Hope Medical Center, Duarte, CA 91010, USA

[2]Department of Hematology, Sir Run Run Shaw Hospital, School of Medicine, Zhejiang University, Hangzhou 310016, China

[3]The Integrative Genomics Core, Beckman Research Institute, City of Hope Medical Center, Duarte, CA 91010, USA

[4]Department of Hematology and Hematopoietic Cell Transplantation (HCT), Beckman Research Institute, City of Hope Medical Center, Duarte, CA 91010, USA

Correspondence: Ling Li, Ph.D. Department of Hematological Malignancies Translational Science, Gehr Family Center for Leukemia Research, Hematologic Malignancies and Stem Cell Transplantation Institute, Beckman Research Institute of City of Hope, Monrovia Research Center 2005, 1500 E. Duarte Rd, Duarte, CA 91010. Tel: 626-218-2011, Fax: 1-626-301-8973, Email: lingli@coh.org.

Materials and methods

Mice

The *Nup98-HoxD13* (*NHD13*) transgenic mice were from Jackson Laboratory. The *Tet2*$^{flox/flox}$ (*Tet2*$^{fl/fl}$) and *Mx1*-Cre mice were mated on a C57BL/6J genetic background. The *NHD13* mice were crossed with *Tet2*$^{fl/fl}$/*Mx1*-Cre or *Tet2*$^{fl/fl}$ mice to generate *NHD13*/*Tet2*$^{fl/fl}$/*Mx1*-Cre and control *NHD13*/*Tet2*$^{fl/fl}$ mice. Both genotypes were injected with poly(I:C) at 6-weeks-old. All mice were drug or test naive and not involved in previous procedures. Mice of the same gender and age were randomly divided into groups. Investigators were blinded to mouse genotype while performing treatment or monitoring engraftment or survival. Mice were bred at the City of Hope (COH) animal facility and received autoclaved water and clean food. All mice were subjected to 12-hour light/dark cycles and kept in controlled ambient room temperature and air humidity conditions. All animal procedures were conducted in accordance with established institutional guidance and approved protocols of the Institutional Animal Care and Use Committee at COH.

Cell line and primary cell culture

The human MDS cell line MDS-L was provided and developed by Dr. Kaoru Tohyama. According to the inventor, cells were cultured in RPMI 1640 medium with 10% FBS and 1% penicillin-streptomycin in the presence of 30 ng/mL human recombinant human IL-3. For MDS-L, no known reference STR profiling is available. To avoid misidentification, cross-contamination, or genetic drifting during the experiments, we replaced the cultured cells with original frozen stock (100 vials totals) which was originally directly provided from inventor Dr. Kaoru Tohyama. Human CB CD34+ and primary MDS CD34+ cells were maintained in StemSpan Serum-Free Expansion Media (StemCell Technologies, Inc.) supplemented with recombinant human SCF (50 ng/mL), Flt3 ligand (100 ng/mL), TPO (100 ng/mL), IL-3 (25 ng/mL), and IL-6 (10 ng/mL).

Flow cytometry

Single cell suspensions from BM, spleen and peripheral blood (PB) were stained with required antibodies for 20 minutes at 4 ℃, washed and analyzed on a 5-laser, 15 detector LSRII. The DAPI (ThermoFisher Scientific)-negative cell population was selected as living cells. Mouse HSPCs were determined by staining with anti-mouse c-kit, Sca-1, CD16/32 and CD34 antibodies, biotin-linked lineage cocktails (including anti-mouse CD3, CD4, CD8, B220, IgM, CD19, CD11B, CD11c, NK1.1, Gr1, CD41 and Ter119 antibodies (eBioscience)) and FITC-labeled streptavidin. The lineage^{-} c-kit^{+} Sca-1^{-} population was defined as LK, and the lineage^{-} c-kit^{+} Sca-1^{+} population as LSK. CMP, GMP and MEP populations were further gated from the LK population based on CD16/32 and CD34 expression. Mouse BM differentiation was evaluated using anti-mouse CD11B, Gr1, B220, CD3 and Ter119 antibodies. Data were analyzed using BD FACS Diva or FlowJo software.

BM c-kit^{+} cell selection

BM c-kit^{+} cells were isolated using mouse CD117 microBeads (Miltenyi Biotec) according to the kit protocol. Briefly, total BM cells were counted and resuspended in MACS buffer. After staining with CD117 microbeads (20μL per 10^{7} total cells, 15 min at 4℃), cells were washed and applied onto MS columns, which were rinsed and placed in a magnetic field. Columns were washed three times with 500 μL buffer and then removed from the separator. Magnetically-labeled cells were flushed out with 1mL buffer. c-kit^{+} cells were counted and used for further experiments.

BM transplantation

BM cells from CD45.2 mutant mice (test cells) and CD45.1 WT mice (support cells) were transplanted intravenously into 6- to 8-week-old CD45.1 recipients irradiated at a dose of 650 cGy. Chimerism of donor-derived hematopoietic cells was monitored by flow cytometry.

RT-qPCR analysis

For RT-qPCR, RNA was isolated using Trizol reagent (Invitrogen) or an RNeasy micro kit (QIAGEN), following standard manufacturers' protocols. cDNA was amplified using a High Capacity cDNA Reverse Transcription Kit (Applied Biosystems). Quantitative real time PCR was performed using TaqMan or SYBR Green master mix (Life Technologies) with 0.2 mM Taqman probe (Life Technologies) or gene-specific primers. Signals were detected with a QuantStudio 7 Flex Real-Time PCR system (Life Biotechnology). Relative expression levels were determined after normalization to GAPDH levels.

Western blotting

Cells were lysed in CytoBuster Protein Extraction Reagent (Millipore), and boiled lysates were then resolved on 7.5%, 10% or 12% SDS-PAGE gels and transferred to nitrocellulose membranes (Bio-Rad). The following primary antibodies were used: TET2 (ab124297) from Abcam, ARIH2 (15006-1-AP) from Proteintech, FGFR1 (9740T), P-STAT5 (9351L), P-AKT (4058S), AKT (9272S), P-ERK1/2 (9272S) and ERK1/2 (4695S) from Cell Signaling Technology, beta-actin (sc-69879), STAT5 (sc-74442) and Ub (sc-8017) from Santa Cruz Biotechnology.

Lentivirus transduction and cell transfection

First, 293T cells were transiently transfected with pCDH plasmids and pMD2G/pSAPX2 packaging plasmids. Then, 24-36 hours later, supernatants containing replication-incompetent lentiviruses were collected and concentrated using PEG-it (System Biosciences). After titration, target cells were exposed to virus (MOI=10), and 48 hours later, cells were sorted by flow cytometry based on GFP/RFP expression.

In vitro colony forming assays

Total BM or c-kit$^+$ cells were obtained and suspended in methylcellulose medium (M3434, StemCell Technologies; colonyGEL 1201, ReachBio Research Labs) and then seeded into plates, according to the manufacturer's protocol. Colonies were counted 7 days later. For replating assays, cells from each plate were harvested and replated at 5×10^3 per well.

Cell viability analysis

Cell viability was determined using the Cell Titer-Glo Luminescent Cell Viability Assay Kit (G7570, Promega). Briefly, cells were cultured under different treatment conditions and transferred to 96-well plates for 30 min. Kit substrate and buffer reagents were then added to each well and mixed to allow cell lysis. Plates were read on a Microplate reader (Beckman culture DTX880). Results were expressed as a percentage of untreated cells, for three replicates.

Whole-exome sequencing (WES)

Initial WES was carried out to identify mutations in gene exomes. Three *NHD13*/*Tet2*$^{fl/fl}$ and five *NHD13*/*Tet2*$^{fl/fl}$/*Mx1*-Cre mice from the same pair of breeders (*NHD13*/*Tet2*$^{fl/fl}$ and *Tet2*$^{fl/fl}$/*Mx1*-Cre) were used to exclude the confounding results of germline mutations. *Tet2* was deleted by injection of poly(I:C) at 6-weeks-old and c-kit$^+$ cells were isolated at 20-weeks-old. Genomic DNA was captured with the Agilent SureSelect all mouse exon probes according to the manufacturer's protocol. 100-bp paired-end sequencing was performed using an Illumina HiSeq 2500 system. Raw sequencing reads were mapped to the whole mouse genome (mm10) using PEMapper/PECaller (https://github.com/wingolab-org/pecaller) with default settings, and variant bases were annotated with SeqAnt (http://seqant.genetics.emory.edu/).

HPRT mutation analysis

The HPRT mutation assay was conducted to evaluate spontaneous forward mutation frequency. Briefly, K562 cells were selected in Hypoxanthine–aminopterin–thymidine (HAT) medium for 3 days to remove pre-existing HPRT mutants. Next, the cells were washed and seeded in normal culture medium for 13 days for mutant expression. After the phenotypic expression period, the cells were added to 96-well microplates (4×10^5 cells/well) in culture medium with selection using the toxic analogue 6-TG (from Sigma UK) at 0.6 μg/ml. Plating efficiency was determined by culturing 200 cells/well in the absence of 6-TG. After 14 days of culturing, colonies of 20+ cells in diameter were counted as viable expansion. The mutation frequency was determined by dividing the number of 6-TG-resistant colonies by the total number of cells plated, normalized by the plating efficiency.

5-hydroxymethylcytosine DNA-IP (hMeDIP) sequencing and analysis

hMeDIP was performed according to a protocol provided in the SimpleDIP Hydroxymethylated DNA IP kit (Cell Signaling Technology Inc.). First, illumina barcode adapters were ligated to sonicated genomic DNA prior to hMeDIP, and DNA was then denatured and incubated with anti-5hmC antibody at 4 ℃ overnight. The DNA/antibody complex was then captured on protein G magnetic beads. Enriched DNA was purified and sequenced followed by standard Illumina protocols on the Illumina HiSeq 2500 platform (Illumina). Sequencing reads were aligned to the mouse genome build mm10 using the Burrows-Wheeler Aligner (BWA, v0.7.5a).

hMeDIP-qPCR analysis

hMeDIP assays were performed based on the manufacturer’s instructions using the SimpleDIP kit. DNA was denatured and IP’d with anti-5hmC or IgG control antibodies and protein G magnetic beads. After three washes, 5hmC-modified DNA was eluted from beads, purified and used as template for qPCR.

Statistical analysis

Data obtained from independent experiments were expressed as means ± SEM, and *n* represented the number of samples. Unless otherwise specified, two-tailed Student’s *t*-tests were used to compare differences between groups. GraphPad Prism 8 software (La Jolla, CA) was used for statistical analyses. $P<0.05$ represented a statistically significant difference.

Sequences of shRNAs

sh*Arih2* #1:

Sense: 5'-CCGGCCCAGGAAGAAGCTGTTTGAACTCGAGTTCAAACAGCTTCTTCCTGGGTTTTTG-3'. Antisense: 5'-AATTCAAAAACCCAGGAAGAAGCTGTTTGAACTCGAGTTCAAACAGCTTCTTCCTGGG-3'.

sh*Arih2* #2:

Sense: 5'- CCGGCGCTACCTCTTTAGGGACTATCTCGAGATAGTCCCTAAAGAGGTAGCGTTTTTG-3'. Antisense: 5'- AATTCAAAAACGCTACCTCTTTAGGGACTATCTCGAGATAGTCCCTAAAGAGGTAGCG-3'.

sh*Setd2* #1:

Sense: 5'- CCGGTTTGACTCTCTGGGATATAATCTCGAGATTATATCCCAGAGAGTCAAATTTTTG-3'. Antisense: 5'-AATTCAAAAATTTGACTCTCTGGGATATAATCTCGAGATTATATCCCAGAGAGTCAAA-3'.

sh*Setd2* #2:

Sense: 5'- CCGGATAGTGTGACCTCGCCTTATTCTCGAGAATAAGGCGAGGTCACACTATTTTTTG-3'. Antisense: 5'- AATTCAAAAAATAGTGTGACCTCGCCTTATTCTCGAGAATAAGGCGAGGTCACACTAT-3'.

sh*Tet3* #1:

Sense: 5'- CCGGTACCTAGACACACCTACTAAGCTCGAGCTTAGTAGGTGTGTCTAGGTATTTTTG-3'. Antisense: 5'- AATTCAAAAATACCTAGACACACCTACTAAGCTCGAGCTTAGTAGGTGTGTCTAGGTA-3'.

sh*Tet3* #2:

Sense: 5'- CCGGCGCCCACAAGGACCAACATAACTCGAGTTATGTTGGTCCTTGTGGGCGTTTTTG-3'. Antisense: 5'- AATTCAAAAACGCCCACAAGGACCAACATAACTCGAGTTATGTTGGTCCTTGTGGGCG-3'.

sh*TET2* #1: Sigma Cat#TRCN0000192770; sh*TET2* #2: Sigma Cat#TRCN0000418976.

Figure S1

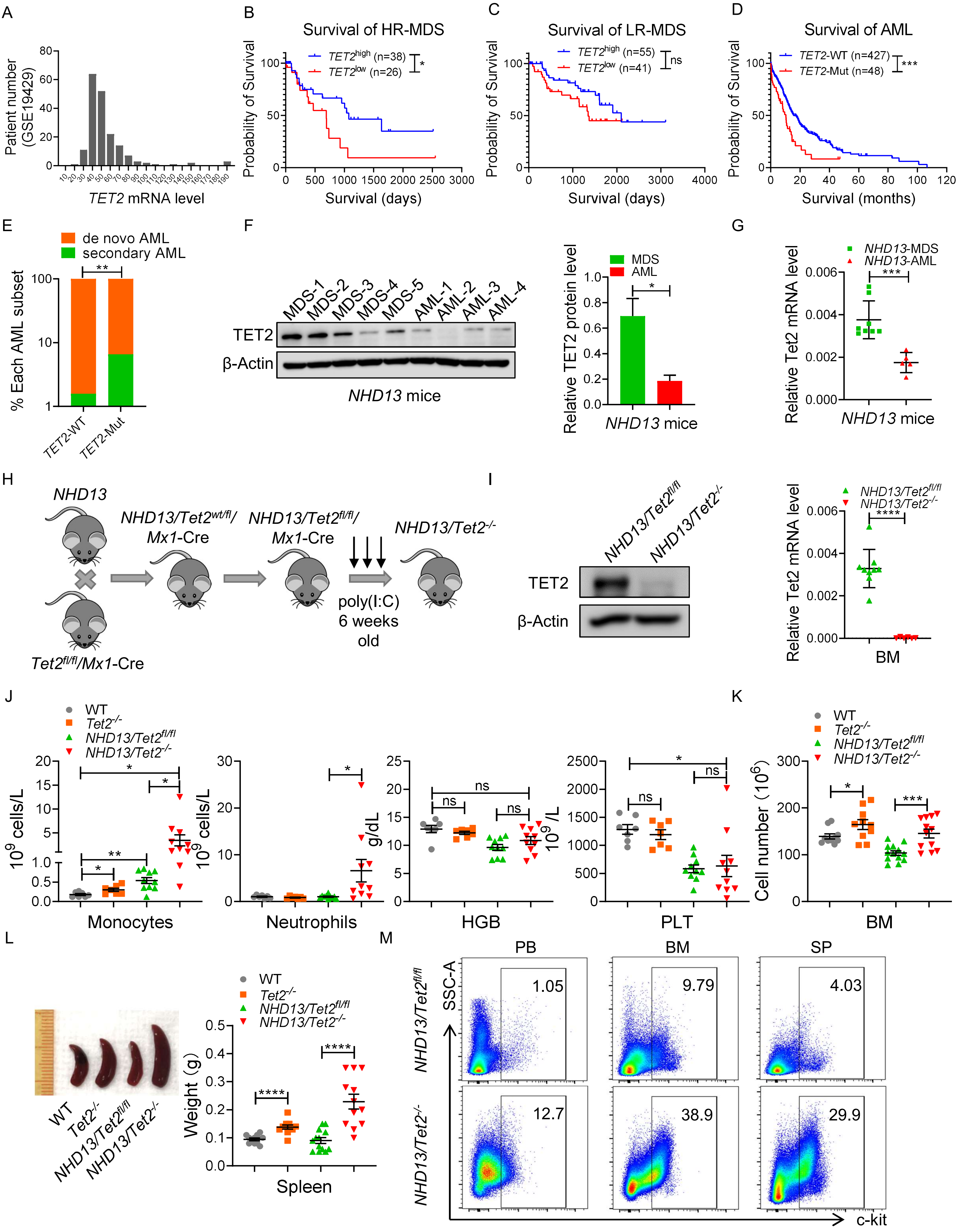
A
Patient number (GSE19429)
TET2 mRNA level
B
Survival of HR-MDS
TET2high (n=38)
TET2low (n=26)
*
Probability of Survival
Survival (days)
C
Survival of LR-MDS
TET2high (n=55)
TET2low (n=41)
ns
Probability of Survival
Survival (days)
D
Survival of AML
TET2-WT (n=427)
TET2-Mut (n=48)
***
Probability of Survival
Survival (months)
E
de novo AML
secondary AML
**
% Each AML subset
TET2-WT
TET2-Mut
F
MDS-1
MDS-2
MDS-3
MDS-4
MDS-5
AML-1
AML-2
AML-3
AML-4
TET2
β-Actin
NHD13 mice
MDS
AML
*
Relative TET2 protein level
NHD13 mice
G
NHD13-MDS
NHD13-AML
***
Relative Tet2 mRNA level
NHD13 mice
H
NHD13
Tet2fl/fl/Mx1-Cre
NHD13/Tet2wt/fl/Mx1-Cre
NHD13/Tet2fl/fl/Mx1-Cre
poly(I:C) 6 weeks old
NHD13/Tet2-/-
I
NHD13/Tet2fl/fl
NHD13/Tet2-/-
TET2
β-Actin
****
Relative Tet2 mRNA level
BM
J
WT
Tet2-/-
NHD13/Tet2fl/fl
NHD13/Tet2-/-
10^9 cells/L
Monocytes
Neutrophils
g/dL
HGB
10^9/L
PLT
ns
K
WT
Tet2-/-
NHD13/Tet2fl/fl
NHD13/Tet2-/-
Cell number (10^6)
BM
L
WT
Tet2-/-
NHD13/Tet2fl/fl
NHD13/Tet2-/-
Weight (g)
Spleen
M
PB
BM
SP
NHD13/Tet2fl/fl
NHD13/Tet2-/-
SSC-A
c-kit
1.05
9.79
4.03
12.7
38.9
29.9

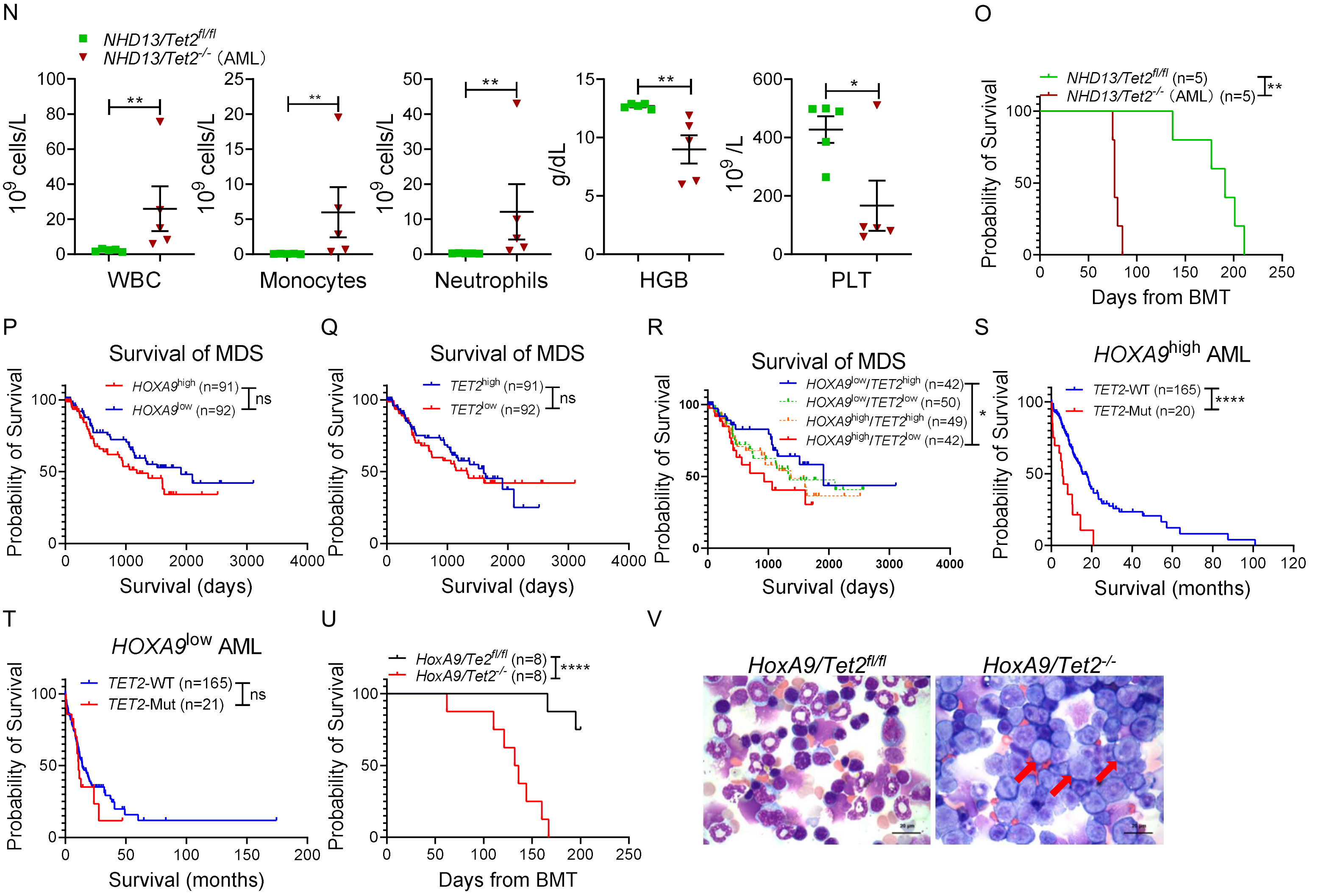

Figure S1. *Tet2* deficiency accelerates leukemia transformation in murine model of MDS.
(A) Histogram of *TET2* mRNA levels in BM CD34⁺ cells from 183 MDS patients (GSE19429). (B and C) Overall survival of patients with high-risk MDS (RAEB I, II cases, termed as HR-MDS, GSE19429) and low-risk MDS (RA and RARS, termed as LR-MDS) stratified by TET2 expression into TET2-high and TET2-low groups. Statistical significance was evaluated by log-rank test. (D) Overall survival of patients with AML (BEAT AML) stratified by TET2 mutation status into TET2-WT (n = 427; median survival, 15.76 months) and TET2-Mut (n = 48; median survival, 10.39 months) groups. (E) Proportion of patients either de novo AML or secondary AML in both *TET2*-WT and -Mutant populations. (F and G) Western blot and q-PCR showing TET2 levels in BM c-kit⁺ cells from *NHD13* mice, as indicated. (H) The scheme shown to generate *NHD13*/*Tet2*-KO mice and further analysis. (I) Western blot and q-PCR showing respective TET2 protein and mRNA levels in total BM cells from indicated mice. (J) CBC analysis of indicated mice at 30 weeks old. (K) Total BM cell number of indicated mice at the leukemia stage. (L) Different size and weight of spleens from WT (n = 11), *Tet2*-KO (n = 11), *NHD13* (n = 13) and *NHD13*/*Tet2*-KO (n = 12) mice at 30 weeks old. (M) Representative c-kit expression profile of PB, BM and spleen (SP) cells isolated from indicated mice at 30 weeks old. (N) CBC of recipient mice receiving BM cells from *NHD13* (n = 5) or *NHD13*/*Tet2*-KO (AML) (n = 5) mice at 8-weeks post-transplantation. (O) Survival of mice receiving BM cells from *NHD13* (n = 5; median survival, 191 days) or *NHD13*/*Tet2*-KO (AML) (n = 5; median survival, 77 days) mice. (P to R) Overall survival of MDS patients (GSE19429) stratified by *HOXA9* and *TET2* expression levels. (S and T) Overall survival of AML patients with *HOXA9*^{high} or *HOXA9*^{low} (BEAT AML) stratified by *TET2* mutation status. (U) Survival of *HoxA9*/*Tet2*-WT (n = 8; median survival, undefined) and *HoxA9*/*Tet2*-KO (n = 8; median survival, 134 days) transplants. (V) Representative Wright Giemsa-stained BM cytospin from *HoxA9*/*Tet2*-WT or *HoxA9*/*Tet2*-KO transplants. Leukemia blast cells are highlighted by red arrows. *P<0.05; **P<0.01; ***P<0.001, ****P<0.0001.

Figure S2

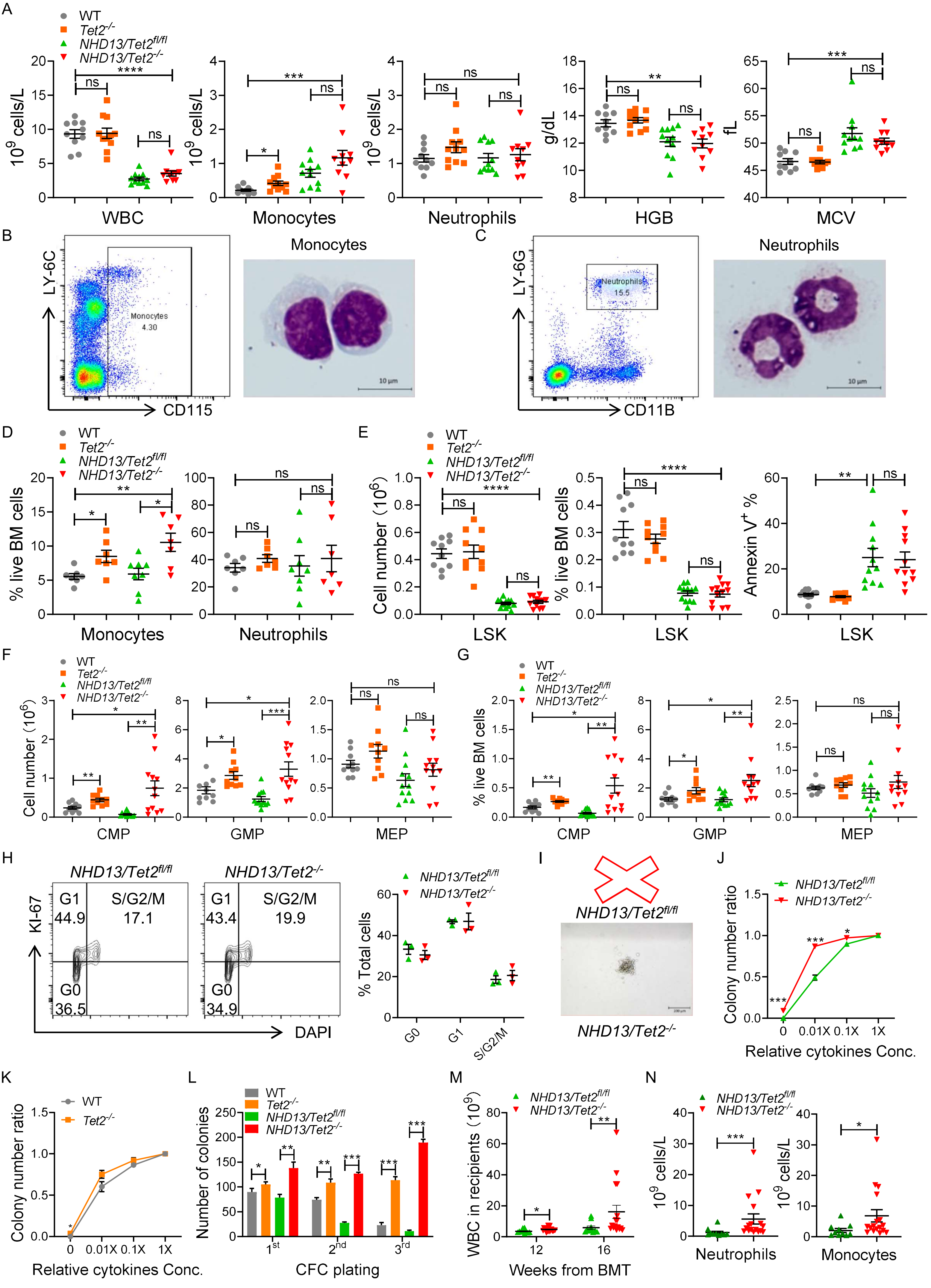
A
WT
Tet2-/-
NHD13/Tet2fl/fl
NHD13/Tet2-/-
10^9 cells/L
WBC
Monocytes
Neutrophils
g/dL
HGB
fL
MCV
ns
*
**
***
****
B
LY-6C
CD115
Monocytes
4.30
Monocytes
10 μm
C
LY-6G
CD11B
Neutrophils
15.5
Neutrophils
10 μm
D
% live BM cells
Monocytes
Neutrophils
E
Cell number (10^6)
LSK
% live BM cells
LSK
Annexin V+ %
LSK
F
Cell number (10^6)
CMP
GMP
MEP
G
% live BM cells
CMP
GMP
MEP
H
NHD13/Tet2fl/fl
NHD13/Tet2-/-
KI-67
DAPI
G1
44.9
S/G2/M
17.1
G0
36.5
G1
43.4
S/G2/M
19.9
G0
34.9
% Total cells
G0
G1
S/G2/M
I
NHD13/Tet2fl/fl
NHD13/Tet2-/-
J
Colony number ratio
Relative cytokines Conc.
0
0.01X
0.1X
1X
K
WT
Tet2-/-
Colony number ratio
Relative cytokines Conc.
L
Number of colonies
CFC plating
1st
2nd
3rd
M
WBC in recipients (10^9)
Weeks from BMT
12
16
N
10^9 cells/L
Neutrophils
Monocytes

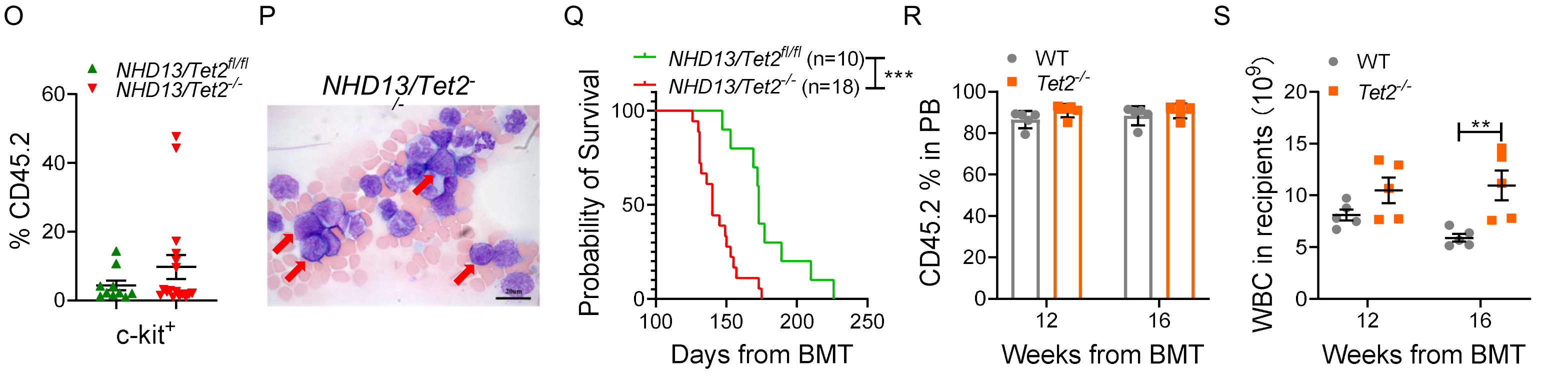

Figure S2. *Tet2* deletion expands the stem/progenitor pool of murine MDS.

(A) CBCs of indicated mice at a pre-leukemic stage (20 weeks old). (B and C) Gating strategy and representative morphology of monocytes (B) and neutrophils (C) in BM. (D) Frequencies of monocytes and neutrophils in BM of indicated primary mice at a pre-leukemic stage. (E) Total cell number, frequency and apoptosis percentage of LSK subsets in BM of indicated primary mice at a pre-leukemic stage (20-weeks-old). (F and G) Numbers (F) and frequencies (G) of CMP, GMP and MEP subsets in BM of indicated primary mice at a pre-leukemic stage. (H) Representative FACS profiles and cumulative results for cell cycle analysis of BM c-kit$^+$ cells from *NHD13*/*Tet2*-WT or *NHD13*/*Tet2*-KO mice. (I) 5 X 10^4 total BM cells isolated from *NHD13*/*Tet2*-WT or *NHD13*/*Tet2*-KO mice at a pre-leukemic stage were plated in duplicate for CFC analysis. Shown are representative morphologies of myeloid colonies grown in the absence of cytokines. (J and K) BM cells isolated from indicated mice were plated in duplicate for CFC analysis, the colony number ratio in each cytokine concentration relative to physiological concentration of cytokines (10ng/mL mIL-3, 10ng/mL mIL-6 and 30ng/mL mSCF) were indicated. (L) Serial replating assays performed using c-kit$^+$ BM cells from indicated primary mice at a pre-leukemic stage. (M to Q) LK cells (2 X 10^5, CD45.2$^+$) from pre-leukemic *NHD13*/*Tet2*-WT or *NHD13*/*Tet2*-KO mice were mixed together with support cells (2 X 10^5 unfractionated BM cells, CD45.1$^+$) from normal control mice and injected into lethally-irradiated recipient mice. (M) WBC in PB of recipient mice at different time points. (N and O) Quantification of neutrophils and monocytes (N) and frequency of c-kit$^+$ cells (O) in PB of recipient mice at 16-weeks post-transplantation. (P) Wright-Giemsa-stained PB smear shows leukemia blast cells (red arrows) in recipient mice that received LK cells from *NHD13*/*Tet2*-KO mice. Scale bars, 20 μm. (Q) Kaplan-Meier comparative survival analysis of *NHD13*/*Tet2*-WT and *NHD13*/*Tet2*-KO BM reconstituted mice. (R and S) c-kit$^+$ cells (2 $\times$ 10^5, CD45.2$^+$) from WT or *Tet2*-KO mice were mixed together with support cells (2 $\times$ 10^5 unfractionated BM cells, CD45.1$^+$) from normal control mice and injected into lethally-irradiated recipient mice. Shown are chimerism of donor-derived cells (CD45.2$^+$) (R) and WBC (S) in PB of WT and *Tet2*-KO recipient mice at different time points.

Figure S3

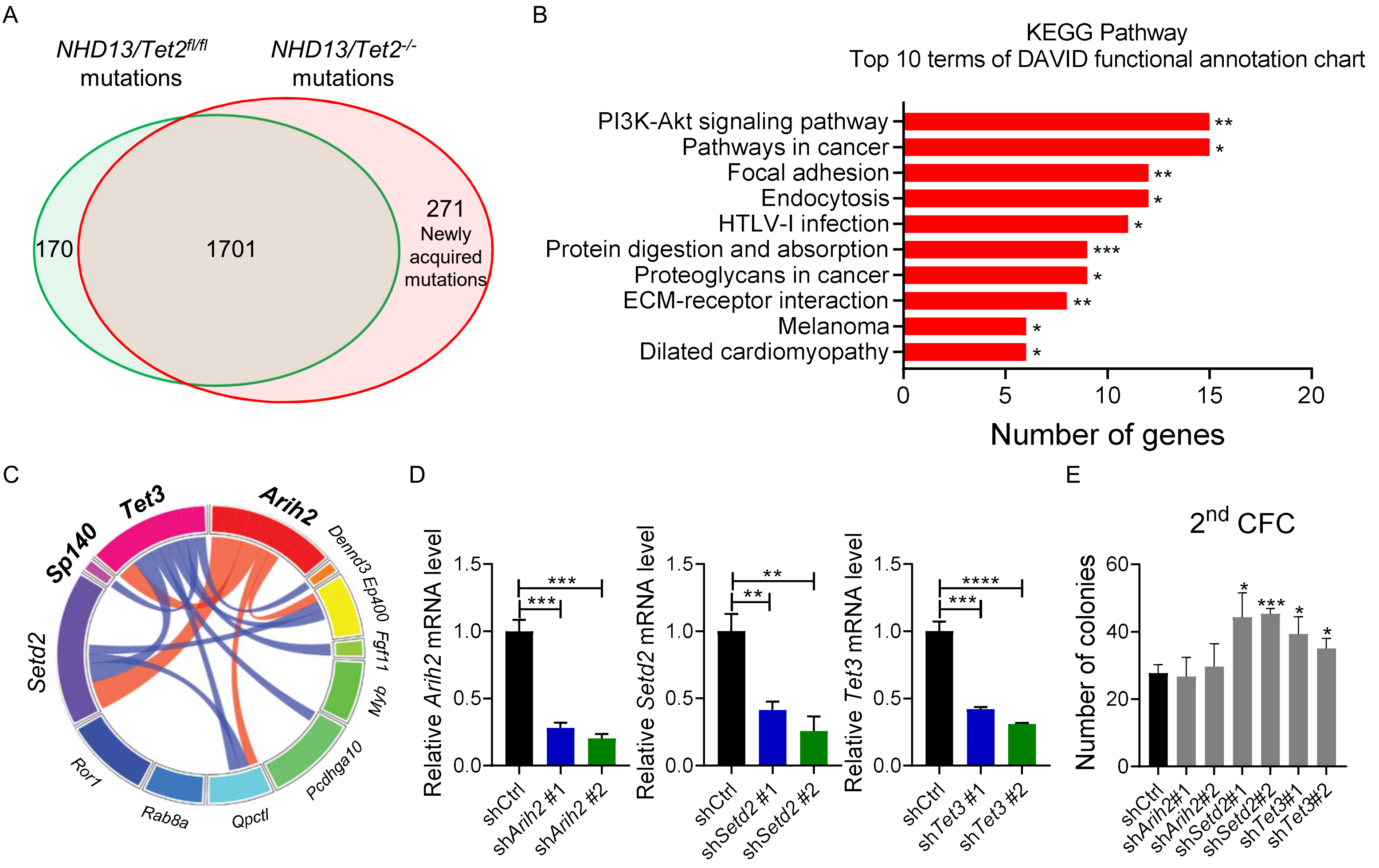

Figure S3. Whole-exome sequencing analysis reveals more genetic alterations in HSPCs from pre-leukemic *NHD13*/*Tet2*-KO mice.

(A) Venn diagram showing overlap of mutations in *NHD13*/*Tet2*-WT and *NHD13*/*Tet2*-KO mice at a pre-leukemic stage, as detected by whole-exome sequencing. (B) KEGG pathway analysis of newly acquired mutations and higher VAF mutations (VAF fold change >1.5) in *NHD13*/*Tet2*-KO mice. Pathways are ranked based on number of enriched genes. (C) Circos plots depicting the relative frequencies and pairwise co-occurrences of selected genetic alterations in *NHD13*/*Tet2*-KO mice. The length of the arc corresponds to the frequency of the first gene mutation, and the width of the ribbon corresponds to the proportion of co-occurrence with the second gene mutation. (D) c-kit$^+$ BM cells from *NHD13* mice were transduced with shRNA against indicated genes, q-PCR showing relative mRNA levels. (E) c-kit$^+$ BM cells from *NHD13* mice were transduced with shRNA targeting indicated genes and then plated for CFC. Shown are colony number after second plating.

Figure S4

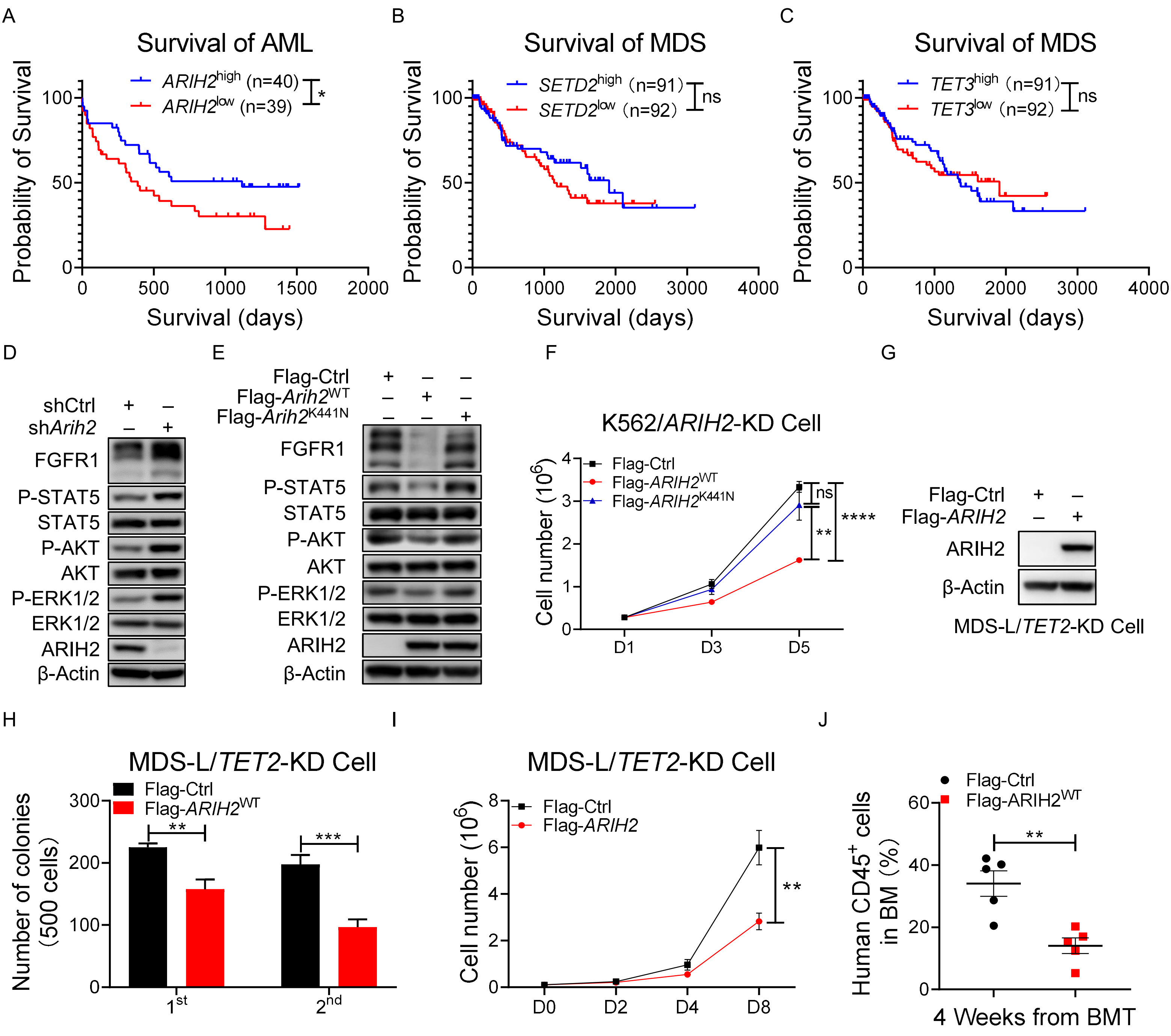

Figure S4. *ARIH2* deficiency promotes MDS cell proliferation.
(A) Overall survival of AML patients (GSE12417) stratified by *ARIH2* expression levels (*ARIH2*^{high}, n = 40, median survival, 1118 days; *ARIH2*^{low}, n = 39, median survival, 388 days) groups. (B and C) Overall survival of MDS patients (GSE19429) stratified by *SETD2* (B) and *TET3* (C) expression. Statistical significance was evaluated by a log-rank test. (D) Western-blot analysis of indicated proteins in K562 cells transduced with either shRNA targeting endogenous *ARIH2* (sh*ARIH2*) or control shRNA (shCtrl). (E and F) *ARIH2*-KD K562 cells were further transduced with empty vector (Flag-Ctrl), Flag-*ARIH2*^{WT} or Flag-*ARIH2*^{K441N}, Western-blot (E) and cell growth (F) were monitored as indicated. Each point is the mean of three determinations with error bars indicating standard deviation. (G) Western blot showing respective *ARIH2* protein level in MDS-L/*TET2*-KD cells overexpressed with full-length *ARIH2* or MOCK controls. (H and I) Full-length *ARIH2* was overexpressed in MDS-L/*TET2*-KD cells, then cells were assessed for CFC replating (H) and cell number during the culture as indicated (I). (J) MDS-L/*TET2*-KD cells transduced with full-length *ARIH2* (Flag-*ARIH2*^{WT}) or MOCK control (Flag-Ctrl) were transplanted into NSGS mice, in-vivo engraftment of human CD45^{+} cells in mouse BM was assessed at 4 weeks post-transplantation.

Figure S5

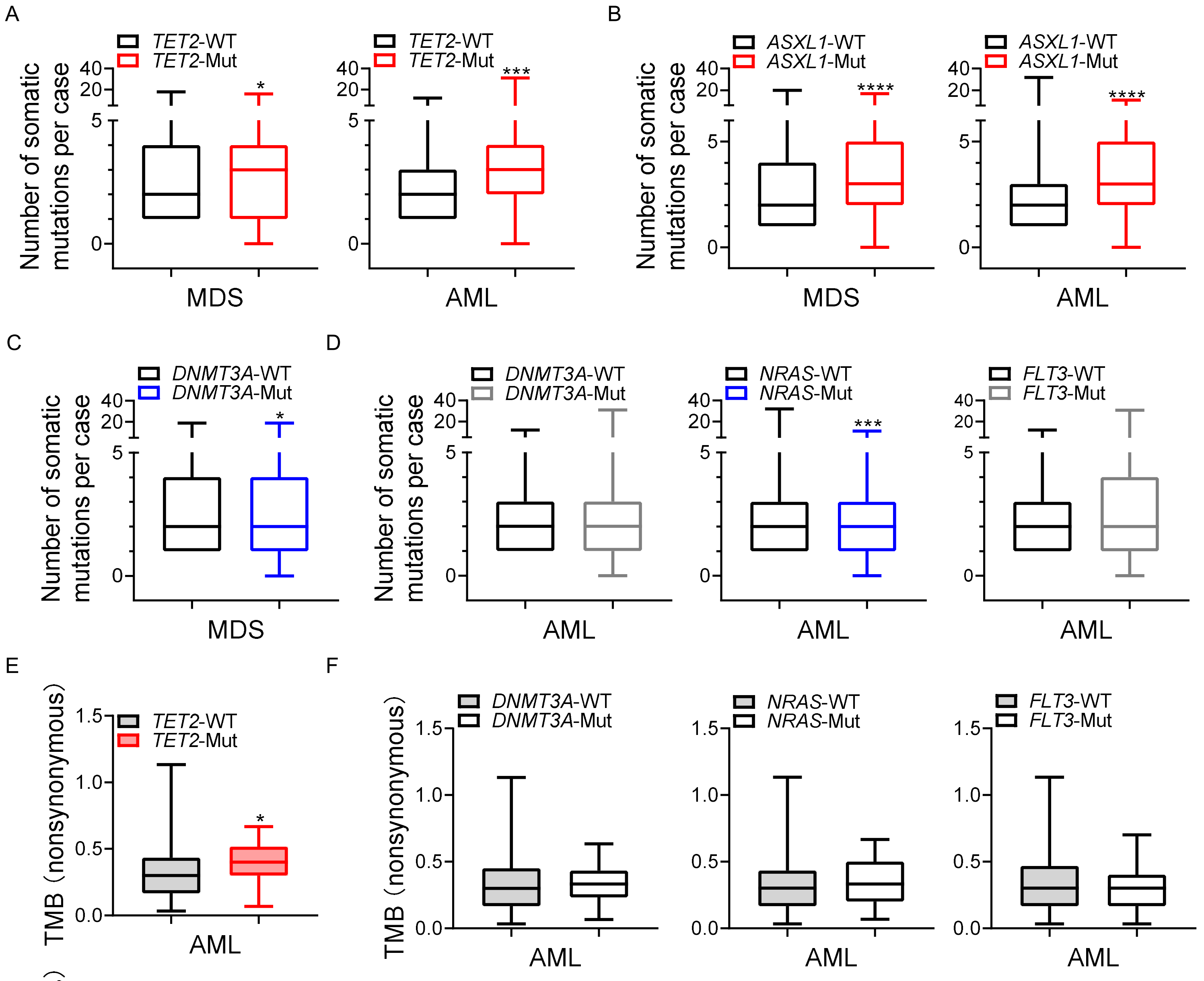

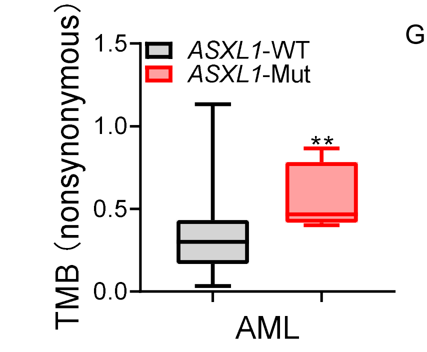

G

K562 cells	Mutation Frequency (10^{-6})	Fold Change	p-value
shCtrl	3.116±0.9251	1	
sh*TET2* #1	59.90±13.24	19.22	0.0129
sh*TET2* #2	71.93±18.57	23.08	0.0208

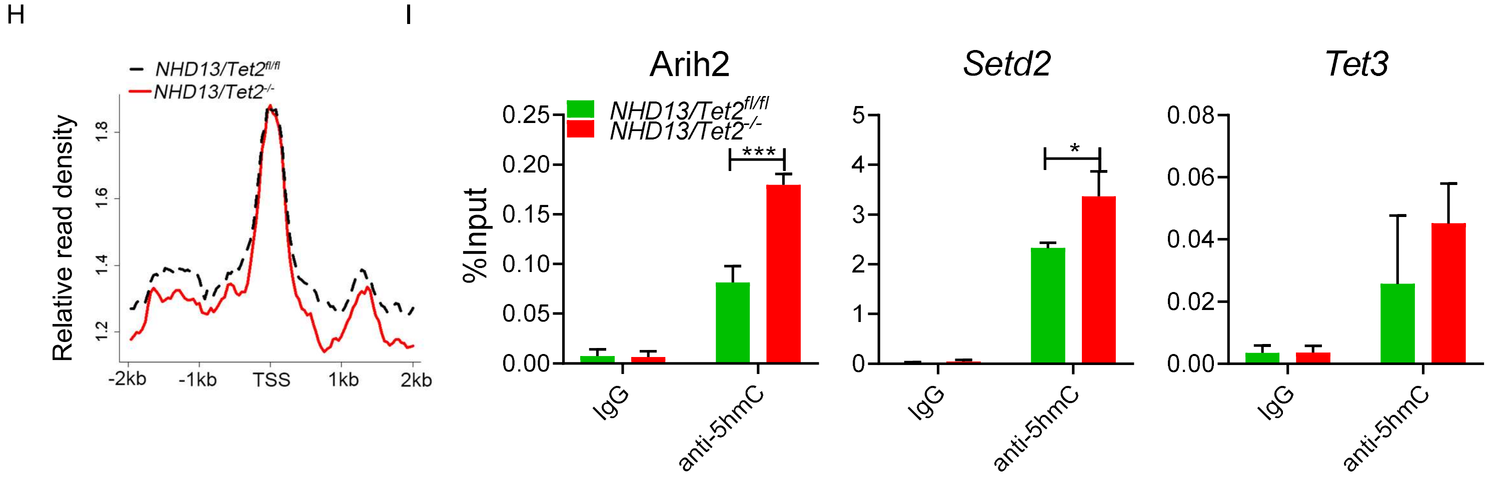

Figure S5. *Tet2* deficiency induces a state of hypermutagenicity.
(A and B) Numbers of nonsynonymous somatic mutations in MDS/AML cases with or without *TET2* (A) or *ASXL1* (B) mutations (Wilcoxon's rank-sum test). (C) Numbers of nonsynonymous somatic mutations in MDS patients with or without *DNMT3A* mutations. (D) Numbers of nonsynonymous somatic mutations in AML patients with or without *DNMT3A*, *NRAS* or *FLT3* mutations. (E and F) Tumor mutational burdens (TMB) in TCGA AML cases with or without indicated mutations. (G) HPRT mutation frequencies in shCtrl or sh*TET2* K562 cells. (H) Distribution of average 5hmC enrichment at all genes in BM c-kit^{+} cells from *Tet2*-WT or *Tet2*-KO *NHD13* mice. (I) hMeDIP-qPCR analysis of 5hmC enrichment at mutant loci of indicated genes in BM c-kit^{+} cells from *Tet2*-WT or *Tet2*-KO *NHD13* mice. Bars represent mean enrichment over input.

Figure S6

A

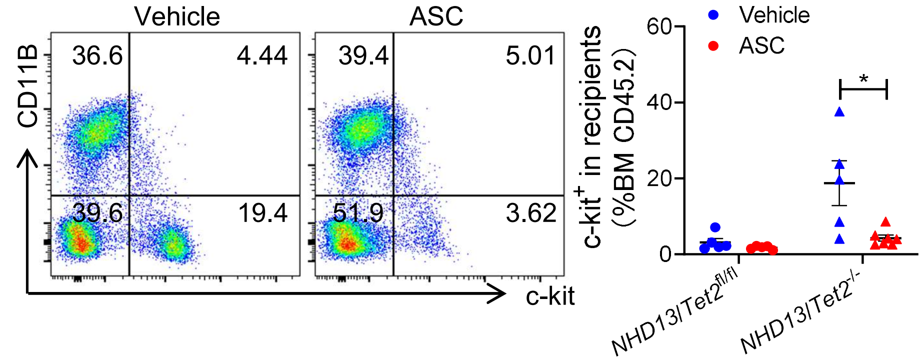

B

Leukemia Transformation	YES	NO	p-value
Vehicle	5	2	
ASC	0	7	0.021

C

K562/*TET2*-KD cells	Mutation Frequency (10^{-6})	Fold Change	p-value
Vehicle	66.23±14.43	1	
ASC	7.338±3.209	0.11	0.0164

D

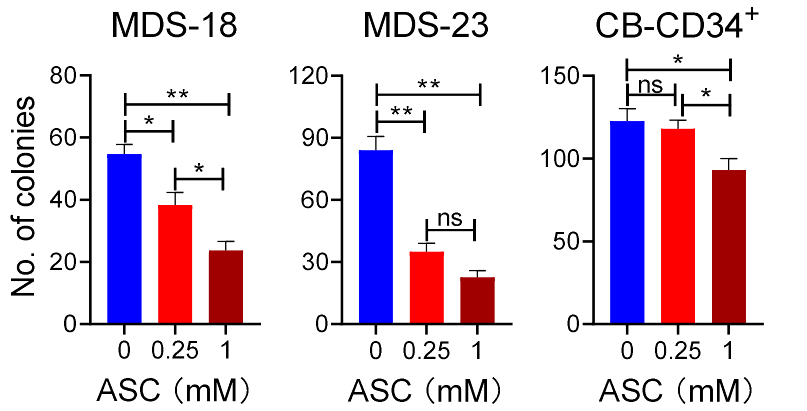

Figure S6. Vitamin C treatment mimics *Tet2* restoration and blocks leukemogenesis.
(A) Vitamin C treatment of mice reconstituted with *NHD13*/*Tet2*$^{fl/fl}$ and *NHD13*/*Tet2*$^{fl/fl}$/*Mx1*-Cre BM. *Tet2* was deleted by injection of poly(I:C) at 6 weeks post-transplant. Mice were intraperitoneally injected with normal saline (vehicle) or ascorbate (ASC, 4 g/kg), 5 days per week for 16 weeks. Expression profile of c-kit^{+} cells in recipient BM were monitored at 24 weeks post-transplant. (B) Leukemia occurrence in vehicle or ASC treated *NHD13*/*Tet2*-KO recipients by 24 weeks post-transplant. (C) K562/*TET2*-KD cells were treated with vehicle or ASC (0.25 mM) for 13 days (during the mutant expression period), HPRT mutation frequencies were evaluated. (D) CFC assay in methylcellulose-containing medium of MDS (MDS-18 and MDS-23, 1 X 10^{5} CD34^{+}) and normal (cord blood [CB], 1 X 10^{4} CD34^{+}) cells treated with ASC at indicated dose for 7 days.

Table S1

ID	Diagnosis	Genotype	Gender	Age (weeks)	Spleen weight (g)	Total BM (10^6)	WBC (10^9)	HGB (g/dL)	MCV （fL）	PLT （10^9）	c-kit% PB	c-kit% SP	c-kit% BM	Giemsa Staining
359	Leukemia	*NHD13*/*Tet2*-KO	M	24	0.27	187.32	40.71	10.5	51.5	249	12.7	38.9	22.9	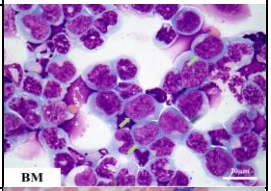
165	Leukemia	*NHD13*/*Tet2*-KO	F	30	1.275	191.35	434.6	9.9	61.1	489	5.2	NA	6.86	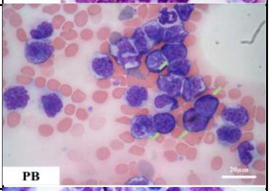
175	Leukemia	*NHD13*/*Tet2*-KO	F	30	0.57	294.35	31.85	10.2	60	1143	3.59	22.1	17.6	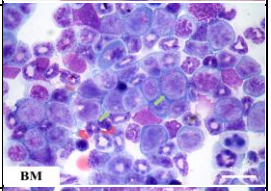
132	Leukemia	*NHD13*/*Tet2*-KO	M	27	0.247	202.32	109.4	8.7	54.6	531	1.07	2.48	0.28	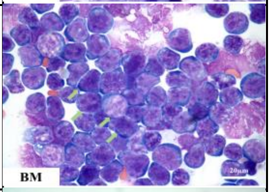
149	Leukemia	*NHD13*/*Tet2*-KO	M	30	0.2	91.35	14.44	7.2	62.1	538	NA	NA	13.3	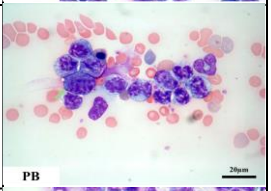
365	MDS	*NHD13*/*Tet2*-KO	M	30	0.15	154.92	6.61	10.9	50.1	619	NA	NA	10.74	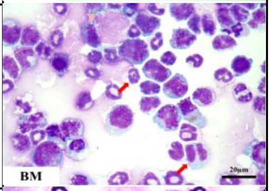
376	MDS	*NHD13*/*Tet2*-KO	M	30	0.15	159.65	3.82	11.4	50.3	618	NA	NA	2.169	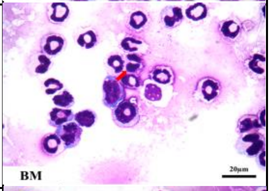
381	MDS	*NHD13*/*Tet2*-KO	M	30	0.13	180.45	2.33	13.3	49.2	1204	NA	NA	4.066	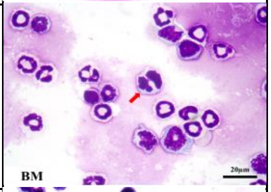
384	MDS	*NHD13*/*Tet2*-KO	F	30	0.08	121.65	3.3	13	51.9	668	NA	NA	2.863	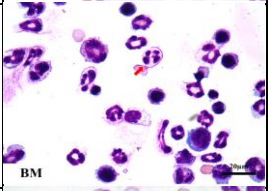
388	MDS	*NHD13*/*Tet2*-KO	M	28	0.35	118.25	4.13	9.3	49.6	2019	NA	NA	4.912	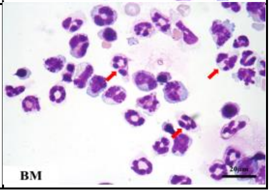

ID	Diagnosis	Genotype	Gender	Age (weeks)	Spleen weight (g)	Total BM (10^6)	WBC (10^9)	HGB (g/dL)	MCV （fL）	PLT （10^9）	c-kit% PB	c-kit% SP	c-kit% BM	Giemsa Staining
49	MDS	*NHD13*	M	30	0.05	102.4	1.88	11.8	52.1	604	NA	NA	1.399	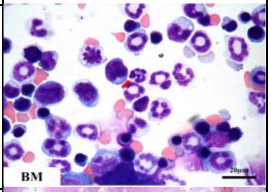
50	MDS	*NHD13*	M	30	0.05	101.85	2.29	11.8	52	563	NA	NA	1.497	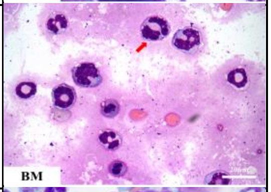
372	MDS	*NHD13*	F	30	0.05	NA	2.18	12.4	55.4	603	NA	NA	2.591	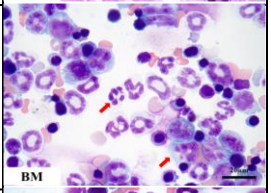
383	MDS	*NHD13*	F	30	0.07	117.8	2.39	14.2	51.4	437	NA	NA	2.834	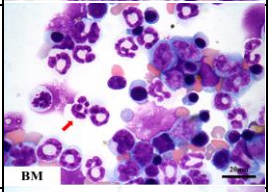
392	MDS	*NHD13*	M	30	0.1	117.1	2.85	9.5	55.2	524	NA	NA	1.018	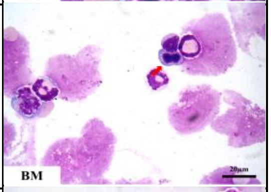
73	MDS	*NHD13*	M	30	0.06	104.4	2.57	10.7	52	842	NA	NA	1.547	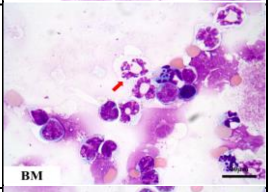
80	MDS	*NHD13*	F	30	0.09	77.8	1.42	11.4	49.7	623	NA	NA	2.324	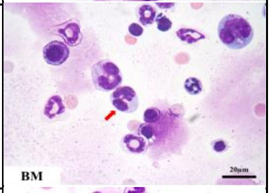
81	MDS	*NHD13*	F	30	0.12	94.25	1.51	11.3	54.1	541	NA	NA	2.271	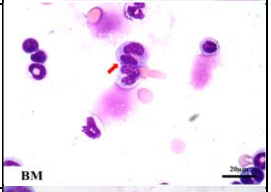
137	MDS	*NHD13*	F	30	0.1	79.75	1.94	11.7	56.2	712	NA	NA	2.389	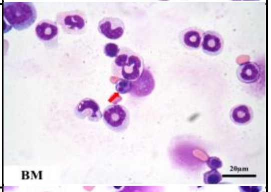
145	MDS	*NHD13*	F	30	0.06	92.2	0.99	7.5	51.2	406	NA	NA	2.551	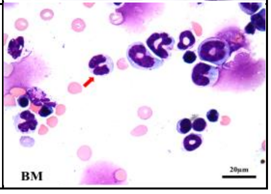

Table S1. *Tet2* loss accelerates leukemogenesis in the *NHD13* mouse model.
Included shows all mice from the survival analyses (Figure 1D). CBC, flow cytometry and morphologic analyses were included in the determination of cause of death. In stained panels, leukemia blasts are indicated by green arrows and dysplastic cells by red.

Table S2

Newly acquired mutations			
Mks1	Wiz	Tsc22d1	Dennd3
Rps19bp1	Abr	Prpf18	Atp13a2
Stx6	Cdk14	Eif3i	Cyp4f13
Astn1	Gm10375	Dnah7b	H2-Q2
Mcm3	Psmc5	Rab5b	1700030J22Rik
Mc3r	Atad2	Lilrb4a	Elp6
Hdgf	9230020A06Rik	Myo1h	Sucla2
Shkbp1	Cngb1	Apc	A430089I19Rik
Mbd5	Tarsl2	Tnfrsf25	Smok3a
Fam83h	Babam1	Nrg2	Qk
Cox4i1	Btbd35f27	Ypel4	Nova2
AA792892	Gabra6	Lonp1	Pkd2
9530091C08Rik	9530077C05Rik	Fstl4	Col9a1
Fer1l4	Gimap4	Cst12	Arap2
Ilkap	Pde5a	Golga1	Pbxip1
Dab2	Tbc1d2	Dip2a	Slc6a6
Nrg1	Fbxo38	Slc12a4	Ift140
Capn11	Selenom	Gm815	Pkn3
Tomm40	A830010M20Rik	Vmn2r116	Pfdn5
Cct6a	Ryr1	Asxl1	Atxn2l
Uba5	Olfr1233	Haus2	Ubr5
Kmt5b	Fhod3	Abcc8	NONE
Fancd2	Col6a5	Lhfpl3	Glb1l
Defb25	Creg2	Mapk8ip2	Asb16
Wasf3	Rapgef4	Zgpat	Rd3l
Zhx1	Kcnt1	Wipf1	Selenow
Lrfn4	Cyp4a12a	Slc8a1	Clcnka
2610301B20Rik	Padi2	Atp4a	Sbf2
Actc1	Arid4b	Pcdhga10	Krt81
Parp3	Dcpp2	9430069I07Rik	Gm10510
Dpp10	Ep400	Col2a1	Erbb3
Prkci	Gm1965	Ablim3	Nkapl
Nrxn2	Zfp462	Il25	Adarb2
Sh2d5	Aldh7a1	Oscp1	Six3
Setd2	Hdac9	Abca13	Gm16573
Mmel1	Ube4b	Pea15a	A730008H23Rik
Rb1	Cdh16	Olfr648	Shtn1
Ptp4a3	Prkch	Ccdc8	Sntb1
Ptpn11	Tmed2	Xpot	Myb
4930452B06Rik	Ifit1bl1	LOC100862268	Zfp446
Grm3	Srp72	Celsr1	Mtch2
Edil3	Acadvl	4930550C14Rik	Spdye4c
Ninj2	Ric8b	Thsd4	Dnm1l
Spata31	Col4a2	Cntnap1	Rnf216
Ugt2b36	Cenpe	Zfp641	Olfr1010
Ube2d2a	Hal	Pex13	Col11a2
Dzip1	Rtn4	Cyp2c68	Tnfrsf11b
Zfand1	Sacm1l	Fgf11	Sstr5
Tmem121	Obsl1	Dyrk1a	Phf1
Spag5	Clasp1	Ngfr	Tspyl4
Tln1	Triobp	Slc2a1	Adamts18
Dnaaf3	Bad	Gmpr2	Fkbp11
Dmwd	Syvn1	Car7	Chd9
Bdp1	Plin2	Wfdc2	Gbgt1
Heg1	Cxcl2	Yjefn3	Lin28a
Wnk2	Araf	Ppargc1a	Bcl7b
Adam5	Otx2	Cntn2	Mrtfa
Olfr20	Plaur	Adam18	Amacr
Mepce	Tmem225	Esrrg	Dpp9
CK137956	Hyou1	Rab8a	Ifit3
Fat3	Zfp207	Gm8765	Samd5
Dlgap2	Gm4884	Lce1b	Qpctl
Sbk1	Pdzrn4	Nkap	Mfge8
Rad51c	Vars2	Neil1	Rab11fip4
Rps17	Slc35g3	Pramef25	Nav1
Clk2	Itga4	Camsap3	Arih2
Stra6	F11	Hmg20b	Bmp10
Tet3	Mir5098	Ubxn2b	

Higher VAF mutations (VAF FC>1.5)		
Hspg2	Sirpb1b	Dnase1l2
Cc2d2a	Thap1	B4galnt3
Igf1	Gm9758	4930579G18Rik
Cmya5	Pam	Erich3
Csmd2	Kcnk16	Gm44502
Csf2ra	Shisa4	Zfp24
G530011O06Rik	Nup210l	Cad
Fbxl5	Vmn2r121	Cyp3a44
Fbxo11	1700049E17Rik1	Tmbim7
Cdk5rap1	Eef2	Polr2a
Radil	Sap130	4933438K21Rik
Vmn1r-ps79	Sp140	Mtor
Gnat2	Pcx	Akap9
Msx2	Car4	Speer4c
Fzd10	Cacna2d2	Slc4a8
Zfp990	Kbtbd11	Nat3
Amz1	Igf1r	Rab3c
Dgka	Acy1	Slc16a14
Tinagl1	Arid1a	Vps13c
Trarg1	Nae1	Mmut
Hebp1	H2-Q7	Asap2
Diaph1	Mep1b	Tbpl1
Gm17019	AU040320	Diaph3
Vmn2r115	Endov	Ccsap
Esm1	Rgl3	Lgals3
Adgrb2	Cct2	Gm10471
Smarca5-ps	Unc13a	Akap13
Ormdl3	Zfp979	Cyp4f18
Speer4d	Tmco6	Trank1
Mcf2l	Muc5b	Arhgap9
Gabarap	Ccdc151	A530032D15Rik
Ecm1	Kif20a	Hoxc4
Ncapd2	Vmn2r88	2900092C05Rik
Rcc2	Slc25a13	Slc26a9
Slc6a9	Cdv3	Amy2a5
Syne3	Th	Vmn2r89
Washc2	Mir148a	Fn3krp
Adamts20	Cenpc1	Bcas3
Speer8-ps1	Thrap3	Ptpru
Ubxn1	6820431F20Rik	Sik1
Thnsl1	Rhbdf2	Nemf
Taf15	Gm15319	Ugt1a1
Lgr6	Myo1c	Hoxa13
Mroh2a	4930572O03Rik	Eya4
Ulk4	Pkd1l2	Cyp3a41a
Ipo9	Atp8a2	Gpatch1
Prdm10	Slc7a8	Bc1
Syt14	Gm13238	2610005L07Rik
Fabp3	Nudt14	Speer4b
Rp1	Rabgap1	Gstcd
Vps8	Speer4a	Itga3
Draxin	Fam102b	Ece1
Gm10354	Slc38a7	Rtn2
Ift122	Dppa5a	Kdm6b
Vmn2r117	Mroh9	Adamts3
Col4a4	Pip5k1b	Adcy2
Poldip2	4930467E23Rik	Terf2ip
Arid3b	Tmprss5	Gm1070
Strn3	Ptprt	Tns3
Calcoco2	Btbd35f3	Gm2016
Kmt2d	Larp1b	Kcnh3
Cpn1	Hdac10	Brms1l
Nupl1	Nell1	C87414
Mllt10	C130026I21Rik	
Dtx1	Lifr	
Trim34b	Dmxl1	
Zfp987	4933416I08Rik	
Rrs1	Gm7609	

Table S2. KEGG pathway analysis of mutations with increased VAF in *NHD13*/*Tet2*-KO mice. 470 genetic alterations with significantly increased VAF (fold change > 1.5) or newly acquired mutations in *NHD13*/*Tet2*-KO mice were analyzed for enrichment in KEGG gene sets using DAVID Functional Annotation Tools. Pathways were ranked based on the number of enriched genes.

Table S3

#CHROM	POS	REF	ALT	Gene Name	Z-score	Survival relevant	Average VAF	Mutation Ratio
chr1	85630543	C	G	*SP140*	-4.5	Positive	0.1191	0.6
chr6	83403661	CCT	C	*TET3*	-3.16		0.5879	0.6
chr7	19081324	G	A	*DMWD*	-2.64		0.7500	0.4
chr18	37747302	A	AAAGG	*PCDHGA10*	-1.96		0.4421	0.4
chr9	60394218	G	C	*THSD4*	-1.92		0.2414	0.2
chr15	73523439	GGT	G	*DENND3*	-1.82		0.0675	0.4
chr8	72180049	G	A	*RAB8A*	-1.62		0.2917	0.2
chr10	21154678	ACTT	A	*MYB*	-1.48		0.3056	0.2
chr7	19143225	C	CG	*QPCTL*	-1.31		0.2812	0.2
chr11	69798282	GGA	G	*FGF11*	-1.24		0.0624	0.4
chr13	21468303	C	G	*NKAPL*	-1.2		0.4681	0.2
chr9	110548233	CAG	C	*SETD2*	-1.1		0.7523	0.6
chr9	15922615	C	G	*FAT3*	-1.09		0.6786	0.4
chr1	63547744	G	A	*ADAM23*	-1.08		0.3529	0.2
chr9	108607291	C	A	*ARIH2*	-0.94		0.6126	0.6
chr2	84849932	A	C	*SLC43A1*	-0.88		0.0777	0.4
chr5	110685333	G	GA	*EP400*	-0.77		0.3077	0.2
chr4	100333756	G	A	*ROR1*	-0.48		0.4118	0.2
chr15	101461017	T	C	*KRT81*	-0.31		0.0511	0.2
chr1	136952050	GA	G	*NR5A2*	-0.29		0.1083	0.6
chr2	76811633	G	A	*TTN*	-0.17		0.3987	0.4
chr9	40148450	A	G	*TMEM225*	-0.13		0.2419	0.2
chr5	104502383	C	T	*PKD2*	-0.02		0.5094	0.4
chr18	35649277	G	A	*MZB1*	0	None	0.5263	0.2
chr4	143949820	G	C	*PRAMEF25*	0		0.3200	0.2
chr17	25492208	C	T	*SSTR5*	0.06	Negetive	0.2143	0.2
chr13	23710640	C	T	*HFE*	0.13		0.5000	0.2
chr5	62748987	C	A	*ARAP2*	0.4		0.4154	0.4
chr1	39623100	G	C	*CREG2*	0.46		0.3235	0.2
chr5	121143073	T	A	*PTPN11*	0.54		0.6596	0.2
chr12	4134424	C	T	*ADCY3*	0.6		1.0000	0.2
chr5	104436910	C	A	*SPP1*	1.04		0.3750	0.2
chr7	118193710	C	T	*SMG1*	1.21		0.5000	0.2
chr7	79141687	C	T	*MFGE8*	1.73		0.4167	0.6
chr17	35666943	GGT	G	*VARS2*	2.72		0.0794	0.4
chr4	140992497	G	A	*ATP13A2*	4.04		0.4545	0.2
chr7	110560221	TA	T	*SBF2*	4.22		0.0847	0.2

Table S3. Top altered genes with AML survival data in PRECOG.
470 genes (newly acquired mutations or higher VAF mutations) in *NHD13*/*Tet2*-KO mice were selected and the top 70 altered genes (VAF FC>2, P<0.05) were imported into PRECOG. Shown are 37 genes with meta-z-scores indicating their AML prognostic significance.

Table S4. Clinical characteristics of MDS patients

**Sample**	MDS18	MDS23
**Age (yrs)**	74	78
**Sex**	M	F
**Subtype**	RAEB-I	RAEB-I
**Cytogenetics**	Trisomy 8	Normal Cytogenetic
**TET2 mutation**	c. 5284A>G (p.I1762V)	c. 2599T>C (p.Y867H)
**WBC**	7.5	14.4
**%Blasts (PB)**	1	3
**% Blasts (BM)**	6	11.2
